# Thrombose bilatérale post-traumatique des artères rénales

**DOI:** 10.11604/pamj.2018.31.192.13908

**Published:** 2018-11-19

**Authors:** Ilyas Derdabi, Hajar El Jouadi, Leila Jroundi

**Affiliations:** 1 Service de Radiologie des Urgences, Hôpital Avicenne, Rabat, Maroc

**Keywords:** Thrombose, traumatisme, vasculaire, rénal, Thrombosis, traumatism, vascular, renal

## Abstract

La thrombose post-traumatique des artères rénales est une complication rarement vue, secondaire à des lésions vasculaires rénales soit par lésion intimale ou compression vertébrale. La conduite diagnostique reste un sujet de discussion compliqué nécessitant la prise en considération de plusieurs facteurs de la part du médecin et du malade.

## Abstract (en)

Posttraumatic bilateral renal artery thrombosis is a rare complication secondary to renovascular lesions including intimal lesion or vertebral compression. Diagnostic approach is a complicated topic of discussions requiring physicians and patients to consider many factors.

## Introduction

La thrombose bilatérale des artères rénales est une urgence post-traumatique rarement décrite dans la littérature. La conduite à tenir reste controversée: la revascularisation chirurgicale immédiate, la néphrectomie et la thérapie conservatrice non opératoire même avec la revascularisation chirurgicale rapide ne permettent pas toujours le retour de la fonction rénale à des valeurs normales.

## Patient et observation

**Figure 1:**
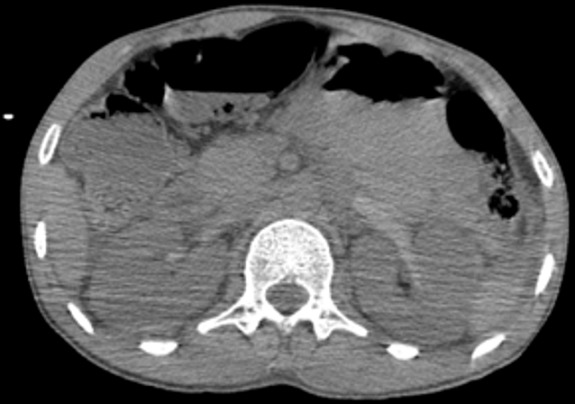
Coupe axiale sans injection: aspect spontanément hyperdense du thrombus de l’artère rénale qui est partiel à droite et total à gauche

**Figure 2:**
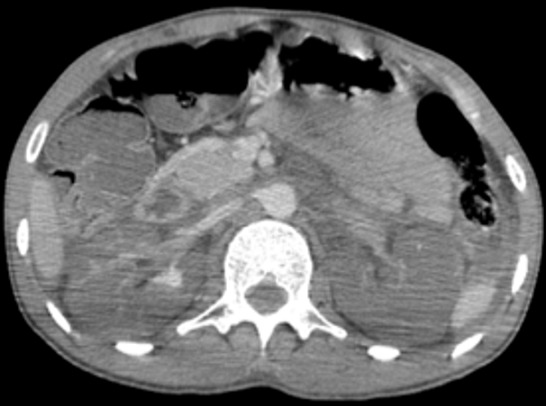
Coupe axiale au temps artériel (rein droit): aspect hypodense du thrombus partiel de l’artère rénale droite avec rehaussement minime de son parenchyme

**Figure 3:**
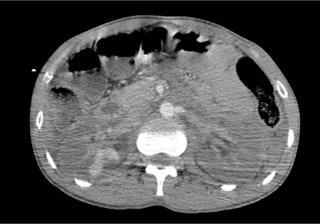
Coupe axiale temps artériel (rein gauche): absence de rehaussement de l’artère rénale gauche ni du parenchyme rénal gauche

**Figure 4:**
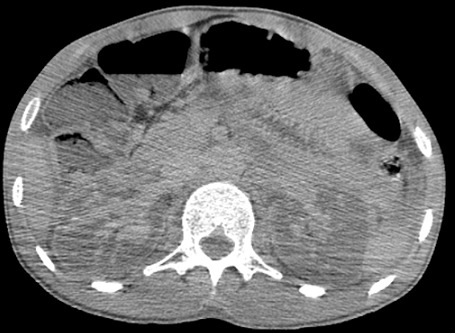
Coupe axiale au temps tardif: absence de sécrétion des deux reins

**Figure 5:**
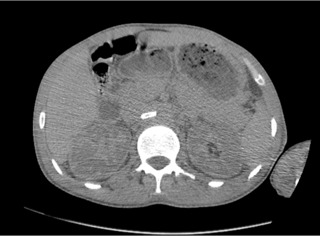
Coupe axiale après pose de Stent: absence de sécrétion des deux reins malgré le Stent

**Figure 6:**
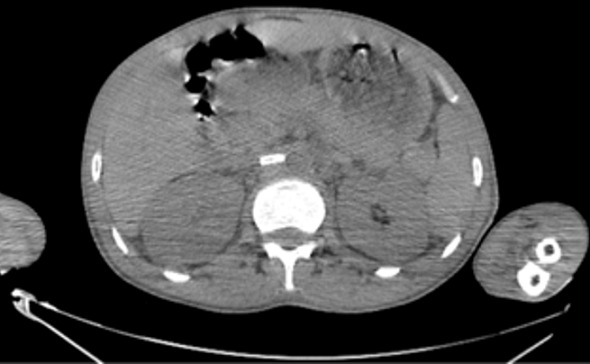
Coupe axiale au temps tardif après pose de Stent: absence d’excrétion des deux côtés

Il s’agit d’un patient de 30 ans, sans antécédent pathologique notable, admis aux urgences pour des douleurs abdominales atroces avec hématurie suite à un accident de la voie publique. Sur le plan clinique, le patient était altéré, avec un score de Glasgow à 10 et des chiffres tensionnels de 90/60, la palpation abdominale a noté une importante défense abdominale. La fonction rénale initiale était normale. Vu l’état du patient, une TDM abdominale avec injection a été réalisée en urgence et qui a montré: deux reins de tailles normales, avec une thrombose partielle de l’artère rénale droite et thrombose totale à gauche, ces thrombus paraissent hyperdenses sur les coupes non injectées (Figure 1), avec un rehaussement minime de la composante parenchymateuse du rein droit dont la thrombose est partielle (Figure 2) et absence de rehaussement total de l’artère rénale gauche dont la thrombose est totale (Figure 3), avec absence de sécrétion à gauche et d’excrétion de façon bilatérale même après un temps tardif de 1h (Figure 4). Une fonction rénale de contrôle a objectivé une créatinine à 100 μmol/l. Le patient est admis au bloc pour éventuel Stent à droite. Le scanner de contrôle a montré une absence de sécrétion (Figure 5) et excrétion malgré le Stent (Figure 6).

## Discussion

Les lésions vasculaires rénales sont une complication bien reconnue des traumatismes abdominaux, elles ont été rapportées chez 1 à 4% des patients suite à un traumatisme abdominal grave. Malgré la rareté relative de ses lésions, l’incidence a augmenté au cours des dernières décennies en raison de l’utilisation facilitée du scanner dans la prise en charge des traumatismes abdominaux [1]. Le mécanisme de survenue de ses lésions a été souvent associé, soit à des phénomènes d’accélération et décélération qui pourraient entrainer des lésions intimales suivies par une dissection ou une thrombose, soit à la compression des artères rénales contre la colonne vertébrale [2]. L’examen physique n’était pas sensible pour le diagnostic de ses lésions et la biologie était non spécifique. La tomodensitométrie était l’examen de choix ayant permis de faire le bilan des lésions et de poser le diagnostique. Ceci est confirmé par des rapports récents qui suggèrent fortement que le diagnostic d’une occlusion de l’artère rénale peut être fait avec une grande précision en utilisant la TDM [3].

La prise en charge de l'occlusion de l'artère rénale traumatique a fait l'objet de controverses, les opinions étant divisées entre une revascularisation chirurgicale immédiate et une observation. Cette controverse a été compliquée par plusieurs facteurs. Premièrement, l'expérience de chaque centre avec ce type de blessure. Deuxièmement, la revascularisation immédiate avait rarement entraîné le retour de la fonction rénale à la normale, comme le cas de notre patient. Troisièmement, le temps d'ischémie prolongée en raison du retard dans le diagnostic, si supérieur à 12 heures, les chances de récupérer la fonction rénale sont sombres. Malgré une amélioration de ce temps, il y avait un manque de corrélation avec la récupération de la fonction rénale. Le temps critique pour sauver le rein peut être plus court que 2-3 heures [4] et souvent ce temps s'écoule avant la revascularisation du rein. Dans la série de Hass *et al.* [5], malgré un temps moyen d'ischémie chaude de 5 heures, seul un patient sur cinq ayant subi une revascularisation chirurgicale a vu sa fonction rénale s’améliorer. Quatrièmement, la majorité des patients présentent d'autres lésions potentiellement mortelles qui les rendent instables. La coagulopathie, l'hypothermie et l'acidose sont la triade mortelle chez ces patients [6]. La prise en charge varie selon l’état du patient, le nombre de lésion uni ou bilatérale et l’état du rein controlatéral. Une approche conservatrice en cas d’atteinte unilatérale avec la présence d'un rein normal controlatéral [7]. Cependant, une approche agressive est indiquée en cas d’occlusion bilatérale de l'artère rénale ou de l'occlusion unilatérale sur rein unique, et ce sans tenir compte du délai [7, 8].

## Conclusion

La thrombose des artères rénales post traumatique est une complication rare et gravissime, nécessitant une prise en charge rapide et adaptée. Les modalités des traitements restent néanmoins controversées avec un taux de réussite faible.

## Conflits d’intérêts

Les auteurs ne déclarent aucun conflit d’intérêts.
